# Promoted hydrogel formation of lignin-containing arabinoxylan aerogel using cellulose nanofibers as a functional biomaterial†

**DOI:** 10.1039/c8ra08166b

**Published:** 2018-11-14

**Authors:** Linn Berglund, Fredrik Forsberg, Mehdi Jonoobi, Kristiina Oksman

**Affiliations:** Division of Materials Science, Luleå University of Technology 97187 Luleå Sweden kristiina.oksman@ltu.se; Division of Fluid and Experimental Mechanics, Luleå University of Technology 97187 Luleå Sweden; Department of Wood and Paper Science and Technology, Faculty of Natural Resources, University of Tehran Karaj Iran; Fibre and Particle Engineering, University of Oulu Oulu Finland

## Abstract

In this work, three-dimensional (3D) aerogels and hydrogels based on lignin-containing arabinoxylan (AX) and cellulose nanofibers (CNF) were prepared. The effects of the CNF and the crosslinking with citric acid (CA) of various contents (1, 3, 5 wt%) were evaluated. All the aerogels possessed highly porous (above 98%) and lightweight structures. The AX-CNF hydrogel with a CA content of 1 wt% revealed a favorable network structure with respect to the swelling ratio; nanofiber addition resulted in a five-fold increase in the degree of swelling (68 g of water per g). The compressive properties were improved when the higher CA content (5 wt%) was used; when combined with CNF, there was a seven-fold enhancement in the compressive strength. The AX-CNF hydrogels were prepared using a green and straightforward method that utilizes sustainable resources efficiently. Therefore, such natural hydrogels could find application potential, for example in the field of soft tissue engineering.

## Abstract 2

Plant biomimicking assembly: the use of cellulose nanofibers as a functional biomaterial for promoted hydrogel formation from lignin-containing arabinoxylan-based aerogels.

## Graphical abstract

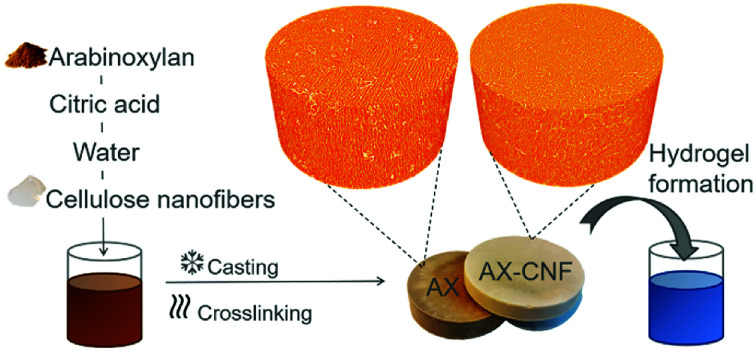

## 1. Introduction

Amongst polysaccharides, cellulose and its nano-derivatives have been extensively utilized to form natural hydrogels.^1^ Research has also increasingly focused on the use of hemicellulose as a promising, low-cost, biodegradable and biocompatible precursor for hydrogels.^2^ Arabinoxylan (AX) is the primary hemicellulose found in annual plants, and is also readily available in residues and side-streams in the agricultural industry.^3^ Furthermore, AX exhibits antioxidant and anti-inflammatory properties because of its ferulic acid content. It also has a vital function in nature, providing rigidity to plant cell walls *via* the crosslinking of polysaccharides and lignin.^4^

Many methods are used to process hydrogels to engineer their properties depending on their intended use; this renders them more attractive for use in a wide range of fields. The large-scale production of packaging materials represents one area where more environmentally friendly products, which allow for biodegradation, are required.^5^ In addition, the biocompatibility of natural structures is beneficial with regard to biomedical applications, such as tissue engineering.^6^

The term aerogel is increasingly used for porous structures dried through different means, such as supercritical drying from alcogel,^7^ or freeze casting *via* lyophilization.^8^ The latter allow for drying directly from water, though both approaches can be applied for the assembly of highly porous, 3D structures that can be used as is, or rehydrated to form a natural hydrogel.

The mechanical robustness of hemicellulose hydrogels have previously shown to be improved by bio-mimicking concepts using cellulose nanofibers (CNFs) as functional and renewable reinforcement material.^8,9^ Another approach involves crosslinking *via* chemical or physical means for structural stability, enabling water uptake without the occurrence of disintegration.^10^ However, to maintain the low environmental impact that the raw material source offers, as well as its biodegradability and biocompatibility, the use of greener, non-toxic crosslinking agents should be considered. Citric acid (CA) is regarded as a non-toxic and relatively inexpensive crosslinking agent that has been used to modify polysaccharides such as cellulose^11^ and xylan.^12,13^

Still, the limited number of available commercial products consisting of natural hydrogels is related, to some extent, to the inadequate mechanical properties of the hydrogels. In addition, these hydrogels have high production costs, and a relatively complex preparation procedure is required to transform the raw material into the final product.^6^

Lignin-containing materials are a promising approach to reduce cost and energy input as well as need of chemical pre-treatments for efficient conversion technologies. The presence of lignin have been associated with enhanced mechanical performance in aqueous environment,^14^ yet also lower hydrophilicity^15^ of natural nanofiber structures.

Part of the complexity for hemicellulose-based materials lies in obtaining high purity *i.e.* low lignin content and low color value hemicellulose.^16^ Still there have been limited research on the feasibility of processing and properties of lignin-containing biomaterials overall and hemicellulose hydrogels in particular.^17,18^ A better understanding of their properties and the role of CNFs on hydrogel formation can offer new insight into green and efficient conversion of hemicellulose-based hydrogels.

In this study, freeze-casted CA crosslinked hydrogels was formed using lignin-containing AX, where water and CNFs were used as a solvent and as reinforcing building blocks, respectively. This provides an alternative route for the preparation of natural, barley-residue-based hydrogels with superior swelling and mechanical behaviors to those formed with AX alone. It is also important to use straightforward and green processing procedures to prepare the product from the raw materials, hence improving the prospects of commercial use. The aim of this study is to understand the role of the CNFs, in relation to the lignin-containing AX, and to study the effect of crosslinking using various amounts of CA, on the structure–property relationships of the hydrogels. This study characterizes the properties of the as-prepared aerogels with respect to their surface characteristics and internal structures. It also characterizes the formation of the hydrogels with regard to their swelling and mechanical behavior.

## 2. Experimental

### 2.1. Materials

#### 2.1.1 Hemicellulose

AX, extracted from barley (Hordeum vulgare) husk, was purchased from Xylophane AB (Gothenburg, Sweden). The molar mass was 34 × 103 g mol^−1^.^19^ This is composed of 48.5 wt% xylose, 11.7 wt% arabinose, and 3.8 wt% glucose, with a xylose to arabinose ratio of 4.15. It also contains 19.6 wt% lignin, 6.8 wt% protein, 4.2 wt% fats, 4.3 wt% ash, and less than 1 wt% starch, as reported by the supplier.

#### 2.1.2 Crosslinking agent

A CA ACS reagent, ≥99.5%, was purchased from Sigma-Aldrich (Saint Louis, USA), with a molecular weight of 192.12 g mol^−1^.

#### 2.1.3 Cellulose nanofibers

**Fig. 1:**
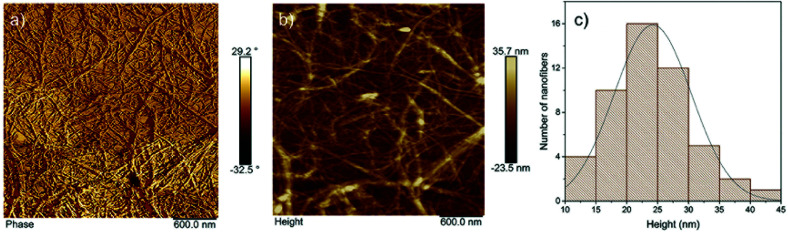
AFM (a) phase and (b) height images of the cellulose nanofibers following the ultrafine grinding process, and (c) size distribution of the nanofiber width, measured from the height image.

CNFs were prepared from brewers' spent grain (BSG), which was obtained following the malting and brewing process from a large Finnish brewery. Purification was performed using a procedure described in a previous study, wherein following the purification process a chemical composition of 70 wt% cellulose, 23 wt% hemicellulose, and 4.2 wt% lignin was reported.^20^ Secondly, the pulp was fibrillated according to a previously reported method.^20^ Briefly, a 2 wt% suspension was fibrillated using a super mass colloider MKCA6-3 (Masuko Sangyo® Co., Ltd. Kawaguchi, Japan), which was operated at 1500 rpm, with a minimum gap of −90 μm. The atomic force microscopy (AFM) phase and height images of the prepared CNFs are shown, together with their size distribution, in Fig. 1. The size distribution was based on the measurements of 50 nanofibers; the average width (height) was 24 ± 6 nm.

### 2.2. Preparation of the aerogels

**Fig. 2:**
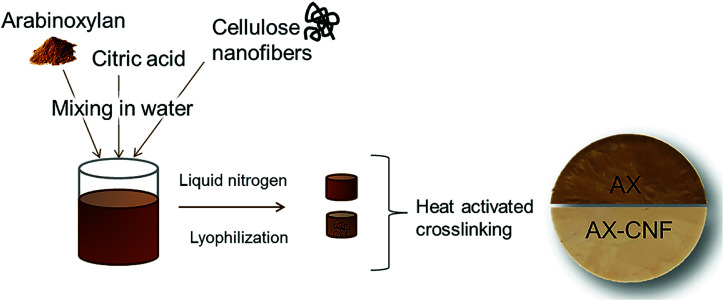
Schematic representation of the procedure used to prepare AX- and AX-CNF aerogels.

The AX and CA were first mixed in distilled water, under magnetic stirring at 30 °C, until the AX dissolved (1 h). The CNFs were added, under continuous stirring, to obtain an AX/CNF ratio of (1 : 1), where the polysaccharide (AX, AX/CNF) content of the distilled water solution was 2 wt%. Various CA contents of 1, 3, and 5 wt% were used. The viscous suspensions were dispersed using a dispersing instrument UltraTurrax, IKA T25, (IKA®-Werke GmbH & Co. KG, Staufen, Germany) operated at 10 000 rpm for 10 min. They were then cast in plastic Petri dishes, where the base of the mold was subsequently exposed to liquid nitrogen. The samples were stored in a freezer at a temperature of approximately −20 °C for at least 24 h prior to lyophilization, which was conducted for 48 h using a freeze-dryer Alpha 2-4 LD plus (CHRIST GmbH, Osterode am Harz, Germany), under a temperature of −40 °C and vacuum of 0.12 mbar. The freeze-casted aerogels were subsequently allowed to react for 3 h within an oven at 120 °C to activate the CA crosslinker. In addition, samples of each composition were maintained at room temperature as control samples with regard to the heat-activated crosslinking reaction. Fig. 2 shows the process used to prepare the aerogel, which was allowed to swell in water to form a hydrogel. In this study, the prepared crosslinked gel-structures are referred to as AX, and the structures with the addition of cellulose nanofibers are denoted as AX-CNF.

### 2.3. Characterization

#### 2.3.1 Density and porosity

The density of the aerogels, denoted as *ρ*_aerogel_, was calculated using a gravimetric method; an analytic balance was used to determine the weight of the aerogels, and a micro-meter gauge was used to measure the thickness of the aerogels, based on an average of ten different measurements. The level of porosity was subsequently calculated using the density value; it was assumed that the skeletal density (*ρ*_cellulose_) of the aerogel was represented by cellulose,^21^ namely 1.46 g cm^−3^:

1 Porosity (%) = 100 × [1 − (*ρ*_aerogel_/*ρ*_cellulose_)]

#### 2.3.2 Swelling properties

The pre-weighed aerogels were immersed in distilled water at room temperature to determine their swelling capacity. The swollen hydrogels were removed from the distilled water, and gently blotted in filter paper to remove any excess water. The hydrogels were then weighed at regular intervals until an equilibrium state was reached; this was achieved after 48 and 56 h for the AX-CNF and AX samples, respectively. The data were measured in triplicate and the average values are reported. The equilibrium swelling ratio was calculated using the following equation:^8^

2 Swelling ratio (g g^−1^) = [(*W*_2_ − *W*_1_)/*W*_1_]

where *W*_1_ and *W*_2_ denote the mass of the sample under the aerogel state and the swollen hydrogel state, respectively.

Wettability of the surface of the aerogel was characterized *via* water contact angle measurements using an EASYDROP contact angle measuring system, which incorporated drop shape analysis control (DSA1) and evaluation software (Krüss GmbH, Hamburg, Germany). A 4 μl drop of water was placed onto the sample, and the instrument software calculated the contact angle, over time, using a sessile drop technique; the reported values are the average of ten measurements.

#### 2.3.3 Mechanical performance

Uniaxial compression tests were performed on the swollen crosslinked hydrogels in their equilibrium state at 25 °C using a dynamic mechanical analyzer DMA Q800 (TA Instruments, New Castle, USA). The samples were compressed up to a strain of 100%, at a strain rate of 10% min^−1^. Prior to each test, the samples were preloaded using a load of 0.05 N. The modulus (E_<5%_) was calculated from the initial linear region of the stress–strain curves with a strain level below 5%. In addition to the maximum compressive strength (*σ*) the materials were compared by the stress (*σ*_50%_) and tangent modulus (E_50%_) at a 50% compressive strain level. For each gel, samples, with dimensions of 15 × 15 mm, and a height of 10 mm were tested eight times for reproducibility reasons; the average results are reported.

#### 2.3.4 Fourier transform infrared (FTIR) analysis

FTIR spectra were recorded using a FTIR spectrometer VERTEX 80 (Bruker Corp., Billerica, USA) to determine any changes in the functional groups of the AX and AX-CNF aerogels (5 wt%). The highest concentration of 5 wt% was chosen in order to amplify the FTIR signals. The absorbance mode was used to analyze the samples over the wavelength range of 4000–400 cm^−1^. For the analysis, a disk was prepared from finely ground samples, which were mixed with KBr, using a pressure of 400 kg cm^−2^ for a period of about 1 min.

#### 2.3.5 Scanning electron microscopy (SEM)

The microstructure of the aerogels fractured surfaces along the freezing direction, as prepared with liquid nitrogen, was observed using a SEM JSM-IT300LV (JEOL®, Tokyo, Japan) at an acceleration voltage of 15 kV. The samples were coated using a coating system (Bal-Tec MED 020) with a tungsten target. The coating was performed within a vacuum of approximately 6 × 10^−5^ mbar, under a current of 100 mA, for 20 s to obtain a coating thickness of 3–5 nm.

X-ray microtomography was used to examine the 3D internal microstructure for two of the aerogels (AX and AX-CNF) at 5 wt% CA content. The samples of approximate size 4 × 4 × 4 mm, were scanned using a Zeiss Xradia 510 Versa (Carl Zeiss, Pleasanton, CA, USA), using a 20× objective, which allowed interior tomography with a field of view (FOV) of 0.66 mm, and the spatial resolution 0.67 μm (voxel size). The scanned region of interest (ROI) was positioned in the exact center of each aerogel sample. The scanning was carried out with an X-ray tube voltage of 60 kV, an output effect of 5 W, and without any X-ray filters. A total number of 2201 projections were acquired with an exposure time of 4 s, which resulted in a total scan time of 3 h and 20 min. The tomographic reconstruction was carried out using filtered backprojection, within Zeiss Scout-and-Scan Reconstructor software (version 11.1). The 3D visualization and analysis of the microstructure in the aerogels were obtained using Dragonfly Pro software (ORS).

## 3. Results and discussion

### 3.1. Porosity of aerogels and their hydrogel formation

The density and porosity of the crosslinked aerogels were measured based on their weight prior to being immersed in water for hydrogel formation. For all samples, porous lightweight structures were obtained after freeze casting and heat activated crosslinking; the average porosity value was 98.4% (Table 1). The average porosity of the samples is comparable to that of hemicellulose-based gels obtained from spruce galactoglucomannans (GGM) and reinforced with wood nanofibers, namely 98.45 ± 0.23% on average.^8^ Furthermore, the values obtained in this study can also be compared to those of cellulose-based gels, namely TEMPO-oxidized wood nanofibers, which were prepared using various solvents prior to sublimation, and have porosity values of 92.8–99%.^21^ The porosity of the hydrogels is regarded a critical parameter with regard to their performance in scaffolds designed for tissue-engineering applications. Here, the highly porous and interconnected microarchitecture provides a large surface area relative to the volume of the scaffold. This promotes cell growth and new tissue formation.^10^ The crosslinking, as well as the addition of CNFs, appeared to have a limited influence on the porosity values; the difference between the highest and lowest values was 0.6%.

**Table 1:** Density and porosity values of the aerogel samples

Sample code	Density (g cm^−3^)	Porosity (%)
AX-1CA	0.023 ± 0.002	98.33 ± 0.00
AX-3CA	0.028 ± 0.002	98.13 ± 0.01
AX-5CA	0.028 ± 0.001	98.13 ± 0.00
AX-1CA-CNF	0.019 ± 0.002	98.73 ± 0.00
AX-3CA-CNF	0.023 ± 0.003	98.47 ± 0.01
AX-5CA-CNF	0.024 ± 0.002	98.40 ± 0.00

**Fig. 3:**
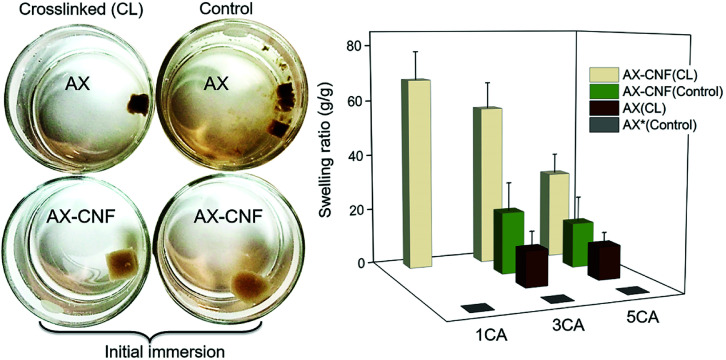
Photograph of the hydrogels with a CA content of 1 wt%, captured immediately following their immersion in water (left). Effect of the CA concentration and CNF addition on the equilibrium swelling ratio (right).

The rehydration of the aerogels for the hydrogel formation and their degree of swelling was the initial assessment in terms of maintaining its structural stability in contact with water, thus reflecting the crosslinking. Both the visual appearance of the samples upon their immersion in water, and the swelling equilibrium of the hydrogels are shown in Fig. 3.

All the prepared hydrogels displayed an opaque color, as shown in Fig. 3. The effect of lignin on hemicellulose extraction has previously been reported by Liu *et al.*^16^ The aforementioned study showed that the presence of lignin limited the dissolution of the hemicellulose and resulted in a relatively dark-colored hemicellulose. This was caused by the linking of the hemicellulose and the formation of lignin-carbohydrate complexes. The AX hydrogels could be visually distinguished because they had a darker color than that of the AX-CNF hydrogels; owing to their greater hemicellulose and lignin contents. When compared with the control samples that were maintained at room temperature, the hydrogels that were placed in an oven after the freeze-casting process, for the heat-activated crosslinking reaction to occur, appeared to have been successfully crosslinked. Thus, the control samples exhibited increased solubility, and consequently, immediately following immersion, lost their structural integrity. This can be observed in Fig. 3 (left). However, the AX hydrogel with a CA content of 1 wt%, which was presumed to have been crosslinked, also disintegrated upon weighing. Consequently, the crosslinked AX-CNF hydrogels were the only samples that were capable of retaining water without their structural stability being compromised at a low CA ratio. At equilibrium, the weight of these samples increased by up to 68 (±10) times their initial weight (Fig. 3, right). This may be attributed to the occurrence of crosslinking between the hydroxyl groups of the cellulose nanofibers and the carboxyl groups of CA,^11^ which further stabilizes the structure, although the crosslinking-agent quantities were low. The degree of swelling of the AX-CNF hydrogels obtained from barley residues was generally greater than those of other nanoreinforced hemicellulose-based hydrogels prepared with comparable techniques, such as liquid-nitrogen followed by lyophilization. Galactoglucomannans (GGM), obtained from spruce and reinforced with nanofibers, absorbed up to 37 g of water per g.^8^ Furthermore, glucuronoarabinoxylan (GAX), obtained from oat spelt and reinforced with nanocrystals, displayed a holding capacity of 25 g of water per g.^22^

As the CA content of the hydrogels increased, a reduction in the degree of swelling was observed for all the hydrogels (Fig. 3); this indicates that there was an increase in the crosslinking density of the samples. This resulted in the presence of a less impenetrable network within the hydrogel, which diminished the swelling ratio.

Interestingly, when the CNF addition was equal to the AX quantity, the structure appeared to be maintained even without the heat-activated crosslinking reaction, as shown in Fig. 3 for the control samples (AX-CNF) with CA contents of 3 and 5 wt%. Moreover, this indicates that the physical bonds formed by the CNFs are not solely responsible for the maintenance of the structures, since the sample with the CA content of 1 wt% lost its shape. Furthermore, this suggests that during processing, the presence of CA, above a content of 3 wt%, has an influence on the structural stability of the sample to some extent. Azeredo *et al.*^23^ indicated that, even at room temperature, the crosslinking reaction occurred within hemicellulose films with CA. Another plausible influencing factor could be owed to the presence of structural irregularities in the form of asymmetrical pores. These are often created during rapid freezing conditions such as that of liquid-nitrogen, and thus undermine stability.^24^

**Fig. 4:**
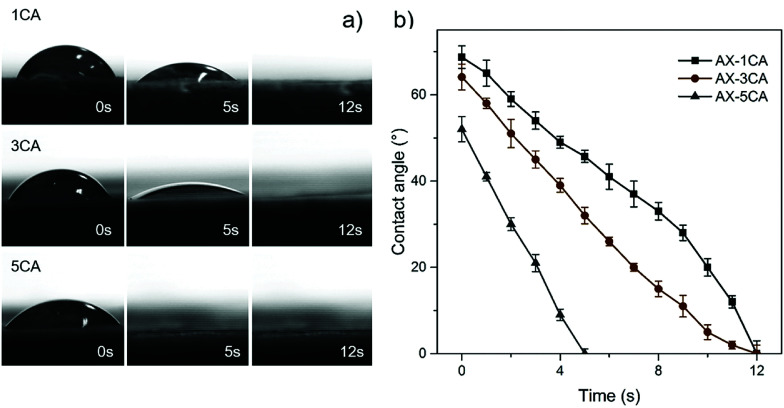
(a) Photographs of the various water adsorption rates of the AX aerogels with CA contents of 1, 3, and 5 wt%, and (b) the measured contact angle, as a function of time, of the AX aerogels with CA contents of 1, 3, and 5 wt%.

The wetting behavior towards water was assessed to further compare the surface water adsorption performance of the various aerogels with respect to the degree of crosslinking and CNF addition. Instant complete wetting behavior was observed for all the AX-CNF aerogels, where the water droplets were adsorbed into the samples in less than one second. Consequently, no contact angles could be measured, with the exception of the AX samples, which showed a more gradual adsorption. This could be partly because the AX aerogels have a greater lignin content, which has a hydrophobic nature, than the AX-CNF aerogels, where hydrophilic cellulose constitutes an equal part of the aerogel. The continuous wetting processes of the AX aerogels with CA contents of 1, 3, and 5 wt% are presented in a series of photographs, shown in Fig. 4a, together with the measured contact angle as a function of time, shown in Fig. 4b.

As shown in Fig. 4a, two wetting regimes were observed during the tests. Initially, good wettability was observed, and over time, complete wetting rapidly occurred for all the AX aerogels irrespective of the CA content. However, the time required to achieve complete wetting appeared to be related to the CA content, where the wettability tended to increase as the quantity of the crosslinking agent increased, as shown in Fig. 4b. Increased hydrophilicity has previously been reported in studies involving the crosslinking of xylan^12^ and cellulose^25^ with CA. This was explained by the incorporation of carboxyl groups on the surface of the material. The contact angle of the AX aerogels decreased as the CA content increased (Fig. 4); this was followed by instant, complete wetting with the CNF addition. These observations indicate that the hydrophilicity of the samples increased, which correlates with the greater equilibrium swelling ratio of the AX-CNF hydrogels, as well as the faster time to obtain equilibrium state.

In the case of the hydrogels, the swelling force is counteracted by the retractive force that is induced by the crosslinked polymer network.^26^ This consequently affects their mechanical behavior. Therefore, the hydrogels that were insoluble in water during the swelling test were assumed to be adequately crosslinked. These samples were further tested under compression.

### 3.2. Mechanical properties of the hydrogels

**Fig. 5:**
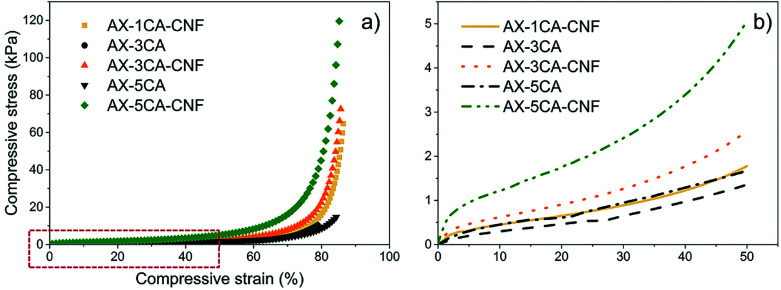
(a) Representative compressive stress–strain curves of the equilibrium swollen AX-CNF hydrogels with CA contents of 1, 3, and 5 wt%, and the AX hydrogels with CA contents of 3 and 5 wt%. *AX_1-CA disintegrated in water, and was not tested in compression. (b) The representative compressive stress–strain curves enlarged at 50% strain.

The compressive stress, as a function of strain, was studied to see the effect of the CNF on the compressive properties of the crosslinked AX hydrogels. The mechanical properties of the hydrogels are displayed in Table 2. The representative compressive stress–strain curves are shown in Fig. 5a, and enlarged for 50% strain level in Fig. 5b.

**Table 2:** Compressive strength (*σ*) and modulus (E_<5%_) determined at the initial portion (strain < 5%) of the stress *versus* strain curves, and stress (*σ*_50%_) and compressive modulus (E_50%_) determined at 50% strain level

Sample code	*σ* (kPa)	E_<5%_ (kPa)	*σ*_50%_ (kPa)	E_50%_ (kPa)
AX-3CA	11.3 ± 4.4	32.6 ± 19.2	1.2 ± 0.6	17.7 ± 2.2
AX-5CA	16.1 ± 3.0	40.9 ± 17.1	1.8 ± 0.4	20.2 ± 2.0
AX-1CA-CNF	67.2 ± 11.2	24.9 ± 13.2	1.5 ± 0.5	4.5 ± 2.4
AX-3CA-CNF	80.7 ± 9.4	33.3 ± 9.0	2.9 ± 0.6	13.2 ± 2.6
AX-5CA-CNF	115.7 ± 6.2	50.4 ± 10.3	4.9 ± 0.4	27.1 ± 3.1

As can be seen in Table 2, the maximum compressive strength (*σ*) of the AX hydrogels with CA contents of 3 and 5 wt% was significantly increased with the CNF addition, representing a seven-fold enhancement, respectively.

The highest strength (116 kPa) of the nanoreinforced AX hydrogel were superior to those reported for a commercially used, synthetic poly(*N*-isopropylacrylamide) (PNIPAM, temperature-responsive polymer) hydrogel reinforced with TEMPO-oxidized CNFs (79.6 kPa), which was tested under similar conditions.^27^ In a previous study, the compression stresses of xylan-rich hemicellulose hydrogels were reported to increase from 13.6 kPa to 38.6 kPa as the degree of crosslinking increased.^28^ This behavior was also observed for both *σ* and *σ*_50%_ of all hydrogels (Table 2 and Fig. 5).

As shown in Fig. 5a, the nanoreinforced hydrogels exhibited “J”-shaped curves, which is typical behavior for soft, biological tissues such as natural tendons and ligaments^29^ and has previously been reported for nanoreinforced hemicellulose-based hydrogels.^30^

To produce soft tissue-engineering scaffolds with sufficient mechanical support, the modulus of the material should lie between 10 kPa and 350 MPa.^31^ For all the hydrogels in this study, the modulus calculated over the linear strain region (Table 2) lie within the lower range of this interval.

**Fig. 6:**
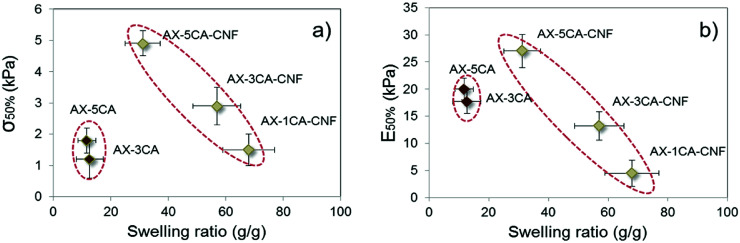
(a) Compressive strength (*σ*_50%_) and (b) compressive modulus (E_50%_) of hydrogels as a function of their swelling ratio.

The correlation between swelling degree and compressive modulus has previously been reported for hemicellulose-reinforced nanofiber hydrogels and explained by the plasticizing effect of water.^9^ The water content has also been reported to strongly affect both compressive strength and modulus of nanoreinforced- polyvinyl alcohol^32^ and gelatin/chitosan^33^ hydrogels negatively. The mechanical properties as a function of swelling degree were thus investigated for the hydrogels in this study at 50% strain, and presented in Fig. 6.

A descending trend was apparent for *σ*_50%_ (Fig. 6a) and E_50%_ (Fig. 6b) of AX-CNF hydrogels with increased swelling. As described in Section 3.1, the reduction in swelling could be explained by the structure stabilization through crosslinking, which in turn was expected to result in enhanced mechanical properties. However, also noticeable from Fig. 6 was that the AX samples were grouped closely together; deviating by their low strength and moderate modulus in relation to their lower swelling ratio. This could be explained by a combination of inadequate crosslinking and the less hydrophilic nature of the lignin-containing AX hydrogels. Inhomogeneous crosslink density distribution, known as spatial inhomogeneity has been reported to dramatically reduce the strength of hydrogels,^34^ this is further in agreement with the significantly lower *σ*_50%_ of AX samples in comparison to their nanoreinforced counterpart at equivalent amount of CA (Fig. 6a).

The hydrophobic nature of lignin combined its natural role as a structural material providing rigidity to plants is likely an influential factor on hydrogel behavior acting to reduce the swelling meanwhile conceivably providing stiffness for the AX samples as seen for E_50%_ in Fig. 6b, this was also apparent from Table 2 for the resistance towards initial pore wall bending of the structure as calculated over the linear region, E_<5%_.

### 3.3. FTIR-analysis

**Fig. 7:**
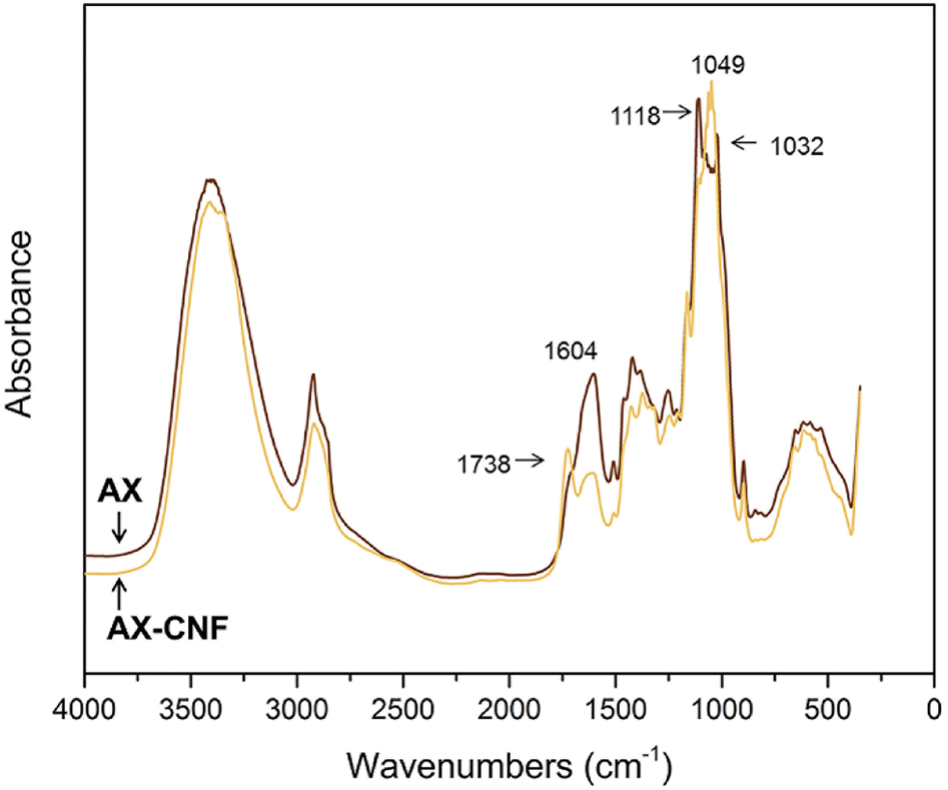
FTIR spectra of the AX and AX-CNF aerogels with CA contents of 5 wt%; obtained following crosslinking.

Following the heat-activated reaction with CA, FTIR spectroscopy was used to compare the AX and AX-CNF samples, as presented in Fig. 7, and study any changes that occurred during crosslinking.

As shown in Fig. 7, the AX sample displays a peak at 1118 cm^−1^. However, in the case of the AX-CNF sample, the peak shifts and is represented at 1049 cm^−1^; both these peaks are reported to be indicative of AX.^35^ In addition, the AX sample also revealed a peak at 1032 cm^−1^, which was assigned to the deformation vibrations of C–H bonds within the aromatic rings associated with lignin.^36^ The peak at 1604 cm^−1^, which is ascribed to the presence of carboxyl groups, was observed in the spectra of both aerogels; however, this peak was more intense in the case of the AX samples (Fig. 7). This peak was previously reported for xylan-based hydrogels that were crosslinked with CA.^13^

A shoulder was observed at around 1738 cm^−1^ in the case of the AX sample; however, a distinct peak appeared at the same position in the case of the AX-CNF aerogel (Fig. 7). This is assigned to the characteristic stretching bands of carbonyl groups of ester groups. It could be owed to the ester linkage of the carboxylic groups of the *p*-coumaric- and ferulic acids of the hemicellulose and/or lignin.^37^ Previous researchers, which observed such signals near 1738 cm^−1^, have also described the peak to be related to anhydride formation. This is an intermediate reaction that is required for the reaction between the CA and hydroxyl groups, and observed during the modification of cellulose^11^ and xylan^12^ with CA contents of 20 wt% or greater. The absence of a distinct peak related to AX may be attributed to the relatively low quantity of CA used in this study.

The results from Fig. 7 indicate that in the presence of the cellulose nanofibers (AX-CNF), the crosslinking of the xylan backbone, *via* the reaction with the CA, appeared to be intensified compared with that of the AX alone. This could be attributed to the three hydroxyl groups of the cellulose, which are available at each monomer unit for esterification. However, the hemicelluloses only possess one or two hydroxyl groups, and thus, they are less susceptible to modification.^35^

### 3.4. Morphology

**Fig. 8:**
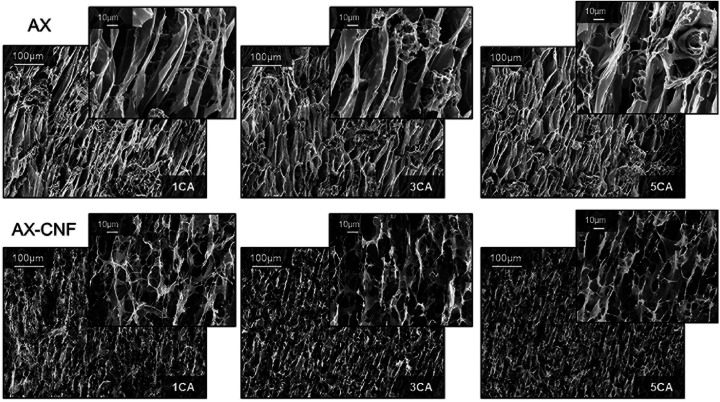
SEM fracture surfaces along the freezing direction of the AX and AX-CNF aerogels with CA contents of 1, 3, and 5 wt%.

The microstructures of the aerogels fractured surfaces in the freezing direction prepared with liquid nitrogen were observed using electron microscope and the micrographs are shown in Fig. 8.

As shown in the top row of Fig. 8, an oriented, channel-like structure dominates the inner architecture of the AX aerogel. During the initial liquid-nitrogen freezing process, ice crystals are formed and grow in the direction parallel to the temperature gradient.^3^ Hence, after sublimation the bottom-up freezing direction of the AX samples displayed an ordered arrangement of elongated channels in the crystal growth direction. The second row of results shows that the formation of a network structure with open interconnected pores was promoted when the CNFs were introduced as a component; this possibly contributed to the superior absorption ability of the sample. However, the crystal growth direction appeared to have been impeded by the introduction of nanofibers, thus the AX-CNF aerogel displayed a less oriented structure, in comparison to that of AX. Orientation of aerogel pores upon freeze casting has previously been described for some polysaccharides, such as AX, glucuronoxylan (GX), glucuronoarabinoxylan (GAX),^3^ guar galactomannan (GM), and galactoxyloglucan (XG).^38^ However, the oriented structure of GAX, as observed with electron microscope was reported to be maintained when cellulose nanocrystals was used as reinforcement^3^

Alakalhunmaa *et al.*^8^ reported the formation of CNFs with oriented lamellar structures during unidirectional freezing, which acted as support pillars within the pore walls, and were thus responsible for the load-bearing ability of the material. This is in agreement with the results of the compression testing in this study; the nanofibers could be responsible for the reinforcement effect, which potentially could be further promoted by their orientation to some extent. When the CA content was increased, there was no obvious variation observed in the scanned images of the AX and AX-CNF, as shown in Fig. 8. This indicates that the aerogel structure that formed during the freeze-casting was not affected by the differences in the CA contents, and also that the structure was maintained throughout the various assembly steps.

**Fig. 9:**
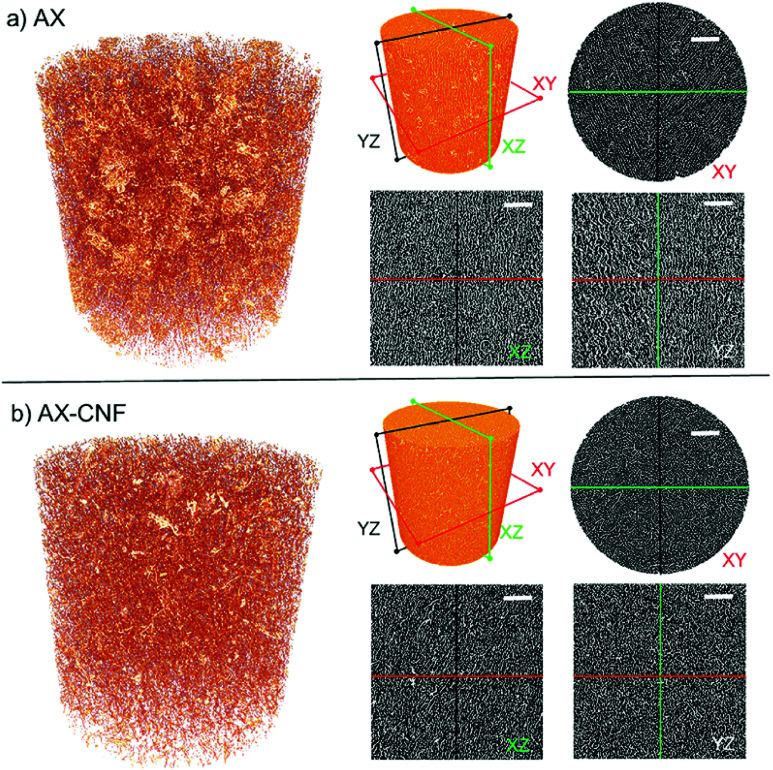
X-ray microtomography 3D reconstruction of the pore structure of (a) AX and (b) AX-CNF and schematic representation of their cross-sectioning for imaging of the aerogels at a CA content of 5 wt% at different sections, respectively. The scale bar corresponds to 100 μm.

The influence of nanofibers on the architectural features was further investigated by X-ray microtomography and presented in Fig. 9. The morphologies seen in two-dimensional microscopy images (Fig. 8) were confirmed from Fig. 9, displaying porous structures. A fibrillar network architecture with the addition of CNFs (b) can be observed in comparison to the clearer channel type structure of AX (a). Furthermore, the preferred growth direction of the ice crystals resulting in oriented pores in the freezing direction for both AX (a) and AX-CNF (b) was confirmed, though the AX-CNF sample showed a less uniform orientation, as observed from the XZ and YZ sections. From Fig. 9, the pores of AX (a) and AX-CNF (b) appeared uniformly distributed in the horizontal (XY), as well as vertical (XZ) and (YZ) sections of the scans. This can be further observed from the 3D reconstruction videos of the samples provided in ESI.1 and ESI.2.†

## 4. Conclusions

In conclusion, highly porous lignin-containing AX-based 3D structures were crosslinked using CA, and reinforced with CNFs. This was achieved by applying a green and robust processing route, which was combined with the effective usage of sustainable resources obtained from inexpensive underutilized barley residues. The compressive properties of the hydrogels were improved by the incorporation of the nanofibers, which resulted in reinforcement and a seven-fold increase in the compressive strength. The CNF addition also promoted the crosslinking, as shown by the FTIR results. The reinforcement effect is believed to have been further promoted by the orientation of the aerogel structure, as shown by the electron microscopy and X-ray microtomography observations. Furthermore, there was a five-fold increase in the degree of swelling; this is likely to be attributed to the formation of a favorable, interconnected network architecture, which is due to the presence of the CNFs.

At low CA contents (1 wt%), the swelling was promoted, where the final weight of the sample was 68 times greater than its initial weight. When the amount of crosslinking agent was increased to 5 wt%, the compressive strength and modulus were improved, but the adsorption performance was impaired. Therefore, the 3 wt% threshold could be used depending on the desired function for the intended application.

The addition of cellulose nanofibers in combination with CA crosslinking greatly promoted the hydrogel formation and properties of AX, thus resulting in an alternative green and efficient processing route of lignin-containing hemicellulose hydrogel that could potentially be used commercially for soft tissue engineering applications.

## Conflicts of interest

There are no conflicts to declare.

## Supplementary Material

RA-008-C8RA08166B-s001

RA-008-C8RA08166B-s002
